# Deciphering the microbiota data from termite mound soil in South Africa using shotgun metagenomics

**DOI:** 10.1016/j.dib.2019.104802

**Published:** 2019-11-13

**Authors:** Ben Jesuorsemwen Enagbonma, Adenike Eunice Amoo, Olubukola Oluranti Babalola

**Affiliations:** Food Security and Safety Niche, Faculty of Natural and Agricultural Sciences, North-West University, Private Mail Bag X2046, Mmabatho, 2735, South Africa

**Keywords:** Illumina miseq, Novel genes, M5NR database, MG-RAST, Metagenomics analysis, Microbial diversity, Soil engineers, Termitarium

## Abstract

We present the metagenomic dataset of the microbial DNA of a termite mound in the North West Province of South Africa. This is the foremost account revealing the microbial diversity of a termite mound soil using the shotgun metagenomics approach in the Province. Next-generation sequencing of the community DNA was carried out on an Illumina Miseq platform. The metagenome comprised of 7,270,818 sequences representing 1,172,099,467 bps with a mean length of 161 bps and 52% G + C content. The sequence data is accessible at the NCBI SRA under the bioproject number PRJNA526912. Metagenomic Rapid Annotations using Subsystems Technology (MG-RAST) was employed for community analysis and it was observed that 0.36% sequences were of archeal origin, 9.51% were eukaryotes and 90.01% were fit to bacteria. A total of 5 archeal, 27 bacterial, and 22 eukaryotic phyla were revealed. Abundant genera were *Sphingomonas* (6.00%), *Streptomyces* (5.00%), *Sphingobium* (4.00%), *Sphingopyxis* (3.00%), and *Mycobacterium* (3.00%), representing 19.23% in the metagenome. For functional examination, Cluster-of-Orthologous-Group (COG) based annotation showed that 46.44% sequences were metabolism associated and 17.45% grouped in the poorly characterized category. Subsystem based annotation method indicated that 14.00% sequences were carbohydrates, 13.00% were clustering-based subsystems, and 10.00% genes for amino acids and derivatives together with the presence of useful traits needed in the body of science.

**Table:** Specifications Table

Subject	Microbiology
Specific subject area	Applied Microbiology and Biotechnology
Type of data	Raw data
How data were acquired	Shotgun metagenome sequencing followed by community and functional metagenome analysis using MG-RAST online server
Data format	FASTQ file
Parameters for data collection	Environmental sample, termiterium, and termite mound soils
Description of data collection	Whole community DNA was extracted from termite mound soil using the PowerSoil® DNA isolation kit. Shotgun metagenomic sequencing was done via the Illumina MiSeq platform
Data source location	Institution: North-West University City/Town/Region: Mafikeng, North West Province Country: South Africa Latitude and longitude (and GPS coordinates) for collected samples/data: 25°26′13.5″S 26°05′50.4“E and (25°27′11.2″S 26°07′33.8″E)
Data accessibility	Repository name NCBI SRA Data identification number: PRJNA526912 Direct URL to data: https://www.ncbi.nlm.nih.gov/bioproject/PRJNA526912

**Table:** 

**Value of the Data** • Profiling the metabolic processes performed by microorganisms is vital both for understanding and for manipulating ecosystems for industrial or research purposes. • Industrial and agricultural biotechnologist. • From this data, there are possibilities of discovering novel genes that may code proteins/enzymes involved in nutrient enhancement, degradation of biomass and control plant pathogen.

## 1 Data description

**Fig. 1:**
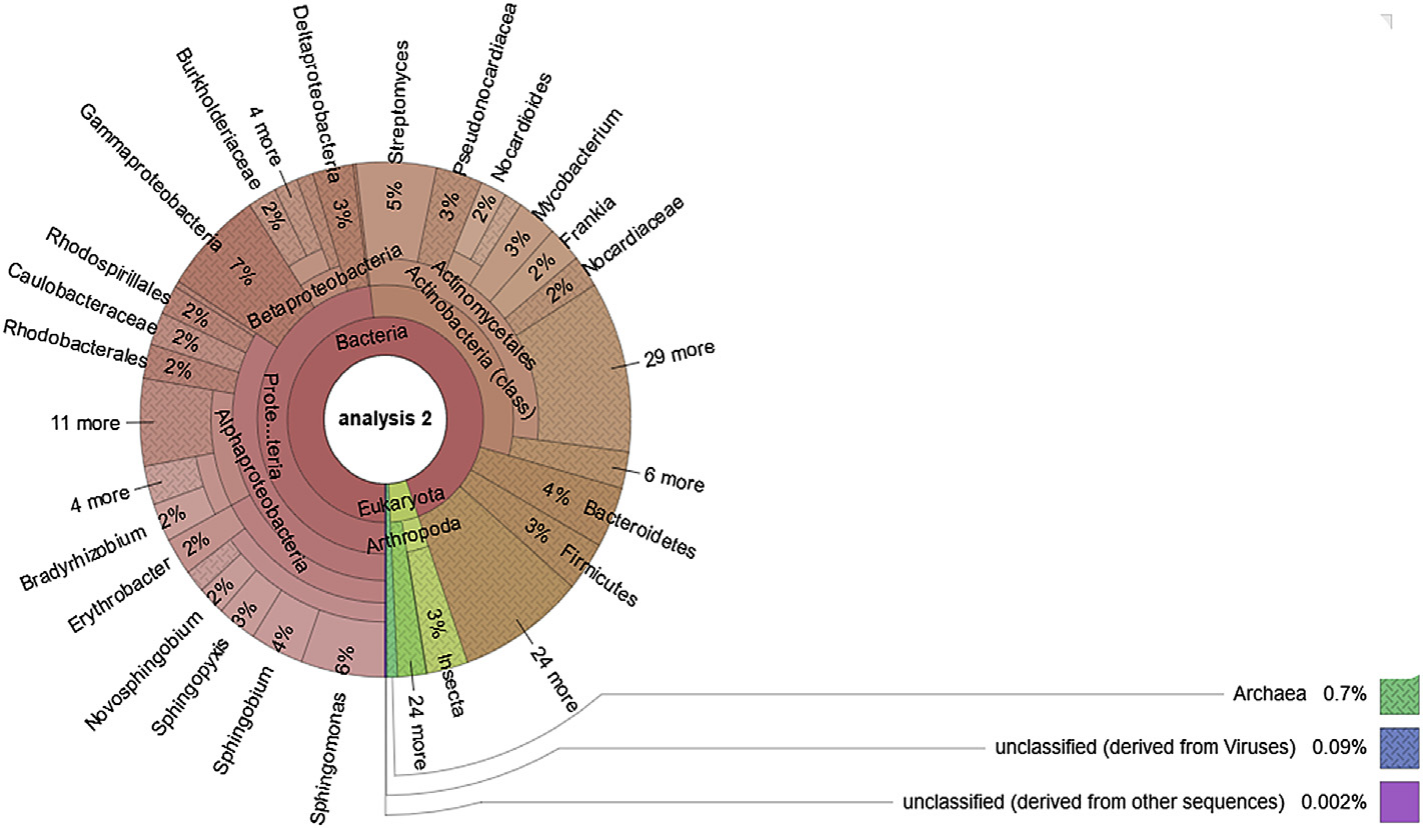
Structure of microbial communities in the termite mound soil metagenome.

**Fig. 2:**
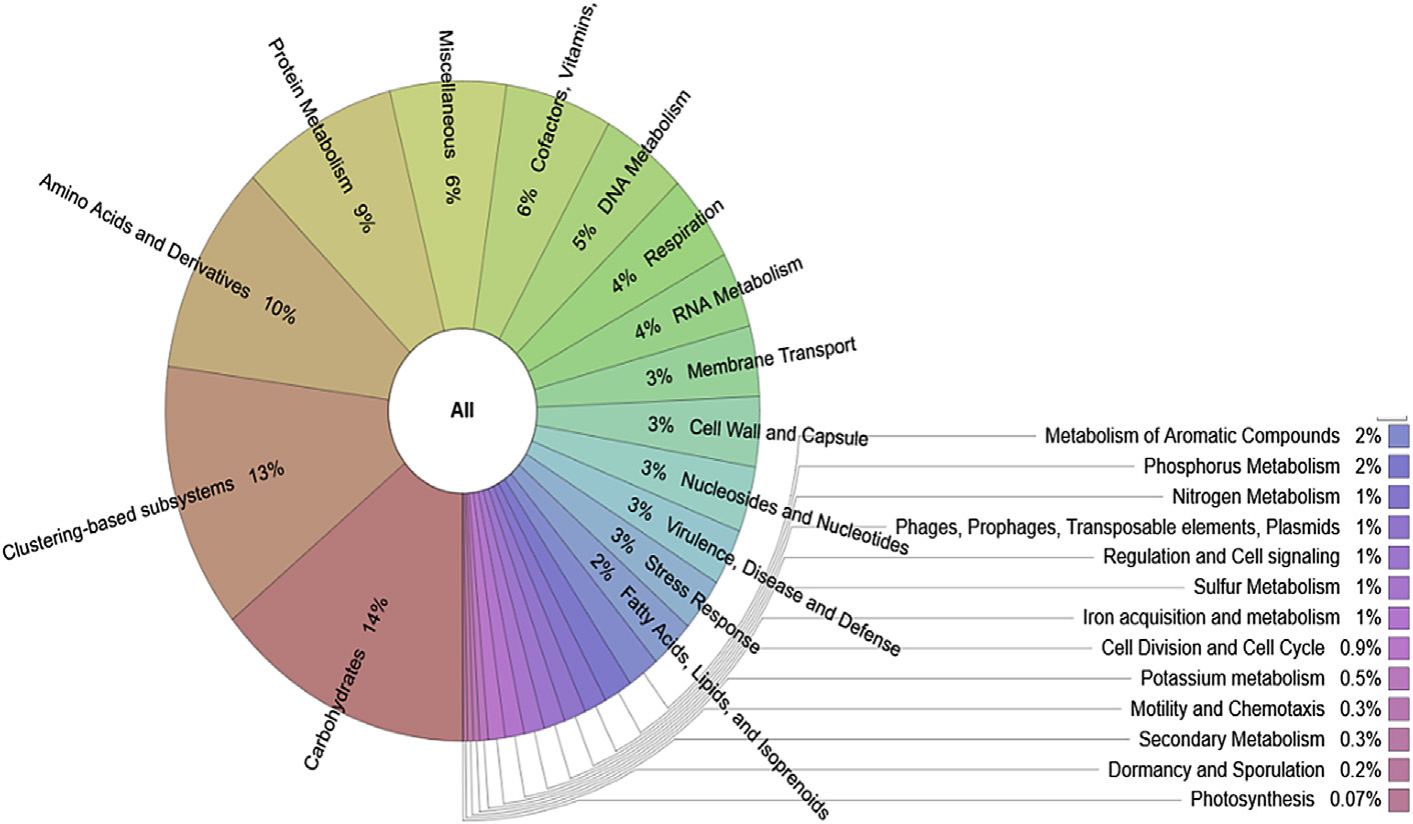
Subsystem-based functional structure of termite mound soil metagenome.

The dataset comprises raw sequencing data acquired through the shotgun sequencing of termite mound soils from North West Province, South Africa. The data files (reads in FASTQ format) were deposited at NCBI SRA database under project accession No. PRJNA526912. Information about the structure of microbial communities and subsystem-based functional structure of termite mound soil metagenome is presented in Fig. 1 and Fig. 2 respectively.

## 2 Experimental design, materials, and methods

In the current dataset, the whole community DNA was extracted from termite mound soils using the PowerSoil® DNA isolation kit (MoBio Laboratories, Incorporation, California, United States of America) following the step by step procedures from the manufacturer's manual. Shotgun metagenomic sequencing was done via the Illumina MiSeq platform according to the manufacturer's guidelines. Analysis and annotation of output data were performed through Metagenomics rapid annotation (MG-RAST) online server [1] with the default parameters. Following quality control (QC), sequences were annotated using BLAT (the BLAST-like alignment tool) algorithm [2], against M5NR database [3] which offers nonredundant integration of numerous databases. Of the 7,270,818 sequences totaling 1,172,099,467 bps with an average length of 161 bps uploaded, 536,311 sequences failed to pass the QC pipeline. While of the sequences that passed QC, 16,693 sequences had ribosomal RNA genes, 2,091,990 sequences contained predicted proteins with known functions, and 3,750,261 sequences had predicted proteins with unknown functions.

## Funding

This work was supported by the National Research Foundation of South Africa [grant numbers UID81192, UID105248, UID95111; OOB]

## References

[1] Meyer F., Paarmann D., D'Souza M., Olson R., Glass E.M., Kubal M., Paczian T., Rodriguez A., Stevens R., Wilke A. (2008). The metagenomics RAST server–a public resource for the automatic phylogenetic and functional analysis of metagenomes. BMC Bioinf..

[2] Kent W.J. (2002). BLAT—the BLAST-like alignment tool. Genome Res..

[3] Wilke A., Harrison T., Wilkening J., Field D., Glass E.M., Kyrpides N., Mavrommatis K., Meyer F. (2012). The M5nr: a novel non-redundant database containing protein sequences and annotations from multiple sources and associated tools. BMC Bioinf..

